# Supplementary material for: Anti-Algics in the Therapeutic Response of Breast and Urological Cancers
Source: Int J Mol Sci. 2023 Dec 29;25(1):468. doi: 10.3390/ijms25010468 (PMC10778606; doi:10.3390/ijms25010468)

Supplementary Materials

Table S1 Selectivity index (SI) values.

Tumoral cell lines	Normal cell lines		
	**Lidocaine**		
	MCF12A (IC_{50}=2,69 mM)	HaCaT (IC_{50}=2,55 mM)	RWPE-1 (IC_{50}=4,35 mM)
MCF-7 (IC_{50}=1,16 mM)	2,3	2,2	*
PC3 (IC_{50}=3,17 mM)	*	*	1,4
LNCaP (IC_{50}=1,74 mM)	*	*	2,5
	**Ropivacaine**		
	MCF12A (IC_{50}=0,86 mM)	HaCaT (IC_{50}=0,90 mM)	RWPE-1(IC_{50} >1 mM)
MCF-7 (IC_{50}=0,49 mM)	1,8	1,8	*
PC3 (IC_{50}=0,99 mM)	*	*	**
LNCaP (IC_{50} >1 mM)	*	*	**
	**Levobupivacaine**		
	MCF12A (IC_{50}=0,40 mM)	HaCaT (IC_{50}=0,64 mM)	RWPE-1 (IC_{50}=0,86 mM)
MCF-7 (IC_{50}=0,16 mM)	2,5	4	*
PC3 (IC_{50}=0,40 mM)	*	*	2,2
LNCaP (IC_{50}=0,56 mM)	*	*	1,5

*Normal cell line is not in respect with tumoral cell line; ** Maximum concentration tested wasn't enough to obtain IC_{50} value

Table S2 Characterization of Opiod group.

Opiod Group, N = 62	
	N (%)
**Opioid**	
Fentanil	34 (54.8)
Tramadol	12 (19.4)
Buprenorfine	10 (16.1)
Tapentadol	5 (8.1)
Hidromorfine	1 (1.6)
Missings	0 (0.0)
**Type of opioid**	
Weak	17 (27.4)
Strong	45 (72.6)
Missings	0 (0.0)
**Exposure time**	
2 months	1 (1.7)
6 months	3 (5.2)
12 months	1 (1.7)
Prolonged	53 (91.4)
Missings	4 (6.5)

**Type of administration**	
Transdermal	43 (69.3)
Oral	19 (30.7)
Missings	0 (0.0)
**Morphine dosage**	
[0, 60]	33 (54.1)
> 60	28 (45.9)
Missings	1 (1.6)

Figure S1. Dotplots of Annexin/PI staining, representing cell viability and cell death profile assessment.

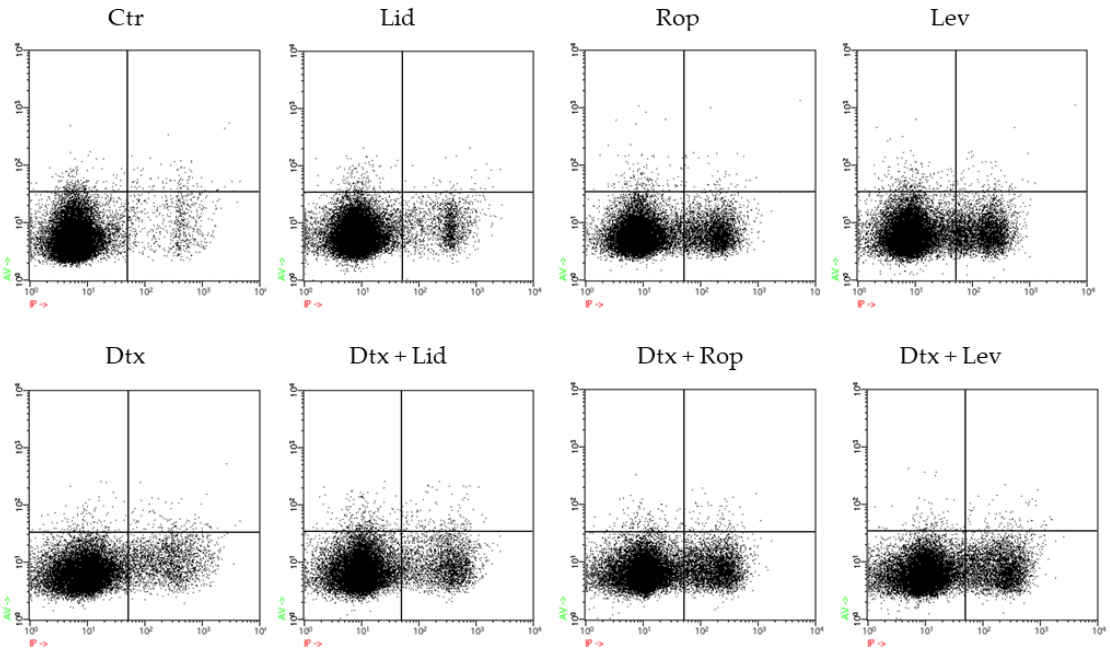

Supplement: Supplementary file 1 [file ijms-25-00468-s001.zip › ijms-2776163-supplementary.pdf]
